# Modeling the Contribution of Allosteric Regulation for Flux Control in the Central Carbon Metabolism of *E. coli*

**DOI:** 10.3389/fbioe.2015.00154

**Published:** 2015-10-08

**Authors:** Daniel Machado, Markus J. Herrgård, Isabel Rocha

**Affiliations:** ^1^Centre of Biological Engineering, University of Minho, Braga, Portugal; ^2^The Novo Nordisk Foundation Center for Biosustainability, Technical University of Denmark, Hørsholm, Denmark

**Keywords:** metabolism, systems biology, constraint-based modeling, allosteric regulation, *Escherichia coli*

## Abstract

Modeling cellular metabolism is fundamental for many biotechnological applications, including drug discovery and rational cell factory design. Central carbon metabolism (CCM) is particularly important as it provides the energy and precursors for other biological processes. However, the complex regulation of CCM pathways has still not been fully unraveled and recent studies have shown that CCM is mostly regulated at post-transcriptional levels. In order to better understand the role of allosteric regulation in controlling the metabolic phenotype, we expand the reconstruction of CCM in *Escherichia coli* with allosteric interactions obtained from relevant databases. This model is used to integrate multi-*omics* datasets and analyze the coordinated changes in enzyme, metabolite, and flux levels between multiple experimental conditions. We observe cases where allosteric interactions have a major contribution to the metabolic flux changes. Inspired by these results, we develop a constraint-based method (arFBA) for simulation of metabolic flux distributions that accounts for allosteric interactions. This method can be used for systematic prediction of potential allosteric regulation under the given experimental conditions based on experimental data. We show that arFBA allows predicting coordinated flux changes that would not be predicted without considering allosteric regulation. The results reveal the importance of key regulatory metabolites, such as *fructose-1,6-bisphosphate*, in controlling the metabolic flux. Accounting for allosteric interactions in metabolic reconstructions reveals a hidden topology in metabolic networks, improving our understanding of cellular metabolism and fostering the development of novel simulation methods that account for this type of regulation.

## 1 Introduction

Mathematical models of metabolism have become a fundamental tool for understanding cellular behavior and for designing genetic or environmental modifications to change that behavior toward a specific purpose (Heinemann and Sauer, 2010). Metabolic models have found applications in both biomedical research and industrial biotechnology. Examples of applications in biomedicine include using metabolic models of human cells to analyze the altered behavior of cancer cells and to suggest potential drug targets (Folger et al., 2011). In the context of industrial biotechnology, models of microbial metabolism are widely used for rational design of microbial cell factories (Zomorrodi et al., 2012).

There are two major approaches for modeling cellular metabolism, namely, kinetic modeling and constraint-based modeling (Machado et al., 2012). The former, based on kinetic rate laws, requires extensive experimental data for determination of the enzymatic mechanisms and respective kinetic parameters. For that reason, these models have been limited to central pathways of well-studied organisms, such as *Escherichia coli* and *Saccharomyces cerevisiae* (Teusink et al., 2000; Chassagnole et al., 2002). Constraint-based modeling, on the other hand, only accounts for the stoichiometry and directionality of biochemical reactions, which can be obtained from genome annotations and limited other information for the organism (Bordbar et al., 2014). With the increasing number of fully sequenced genomes for multiple organisms, the number of genome-scale metabolic reconstructions suitable for constraint-based modeling is also rapidly increasing, with over a hundred reconstructions currently available (Monk et al., 2014).

Constraint-based models can be used to estimate the steady-state flux distribution of a metabolic network, using the so-called Flux Balance Analysis (FBA) approach (Orth et al., 2010). Since the flux solution is not unique with only stoichiometric and directionality constraints, in FBA a single solution is selected based on the assumption of an evolutionary principle of optimality, such as maximization of cellular growth. Methods have been developed to refine metabolic flux predictions by integration of metabolic models with models of other biological processes, such as signaling and transcriptional regulatory networks (Gonçalves et al., 2013). However, some limitations of these methods, such as the reduction of gene expression levels to Boolean states, hamper the predictive ability of the integrated models. More recently, several approaches were developed to directly integrate gene expression data into metabolic models. These methods are based on the assumption that reaction fluxes should be proportional to their respective gene expression levels. However, a recent systematic evaluation of these methods showed little improvement in simulation accuracy when gene or protein expression data are used for flux prediction with a wide range of proposed methods (Machado and Herrgård, 2014). One of the conclusions from this study is that the assumption of proportionality between gene expression levels and reaction rates is not valid for many reactions.

The conclusion that transcriptional or translational regulation does not significantly regulate metabolic fluxes is consistent with recent experimental observations in multiple organisms showing that central carbon metabolism is mostly regulated at post-transcriptional levels (Daran-Lapujade et al., 2007; Chubukov et al., 2013; Kochanowski et al., 2013a). Regulation analysis is a method introduced by ter Kuile and Westerhoff (2001) for quantitatively decomposing flux regulation into *hierarchical* and metabolic coefficients. The former accounts for transcriptional and translational regulation as well as post-translational modifications, whereas the latter accounts for allosteric regulation and thermodynamics. The application of this method to three parasitic protists showed that regulation of glycolytic fluxes is never completely hierarchical, being mostly metabolic in many cases. Similar conclusions were obtained by applying this method to *S. cerevisiae*, where it was observed that metabolic regulation contributed to 50–80% of the flux change in glycolytic enzymes for the given cultivation conditions (Daran-Lapujade et al., 2007).

The partial contribution of transcriptional regulation for flux control in central carbon metabolism can be explained by the cellular trade-off between lowering the investment of protein synthesis (keeping enzymes saturated), and the need to achieve fast regulatory responses and maintain metabolic homeostasis under environmental changes (Fendt et al., 2010; Wessely et al., 2011). In fact, metabolite measurements in *E. coli* and *S. cerevisiae* have shown that most enzymes in central carbon metabolism are not saturated, with substrate levels being close to their respective *K_M_* values (Bennett et al., 2009; Fendt et al., 2010). A recent study in *B. subtilis* showed that transcriptional regulation is insufficient to explain the observed flux change for growth in different carbon sources (Chubukov et al., 2013). Interestingly, the authors observed that the changes in substrate concentrations were also insufficient to explain the observed flux change, leaving an important contribution for post-translational modifications and allosteric regulation.

Learning how allosteric regulation controls the metabolic flux is fundamental for understanding cellular metabolism. Given the growing scope of the constraint-based modeling approach, we propose to expand this formalism with an explicit representation for allosteric interactions. In this work, we build a constraint-based model of allosteric regulation in the central carbon metabolism of *E. coli* and use it to analyze the role of this type of regulation for controlling the metabolic flux under different perturbations.

Allosteric information data are collected from relevant databases and used to build a constraint-based model expanded with allosteric interactions. We analyze how this new layer of interactions affects the network topology in terms of node connectivity and identify relevant metabolic hubs. The model is used as a scaffold to perform regulation analysis using multiple omics data for *E. coli*. Finally, a new method for constraint-based simulation accounting for allosteric interactions is proposed and used for model-based prediction of regulatory effects on flux control.

## 2 Results

### 2.1 Model reconstruction

**Figure 1:**
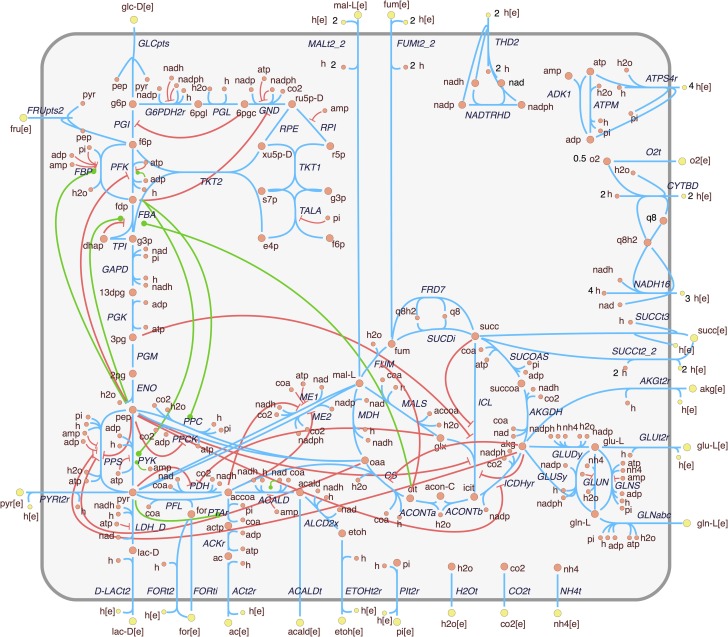
**Extension of the *E. coli* core metabolism model with allosteric interactions**. Enzyme activations and inhibitions are represented, respectively, by green edges with circle ends and red edges with bar ends. This figure is adapted from the metabolic map available at the BiGG database (Schellenberger et al., 2010).

In order to analyze the effects of allosteric regulation in the central carbon metabolism, we expanded a constraint-based model of the core metabolism of *E. coli* (Orth et al., 2009) with allosteric interactions obtained from relevant sources (see Figure S3 in Supplementary Material and Methods section for details). The expanded model is presented in Figure 1. It can be observed that the integration of regulatory interactions reveals an intricate topology that is not captured by the stoichiometric reconstruction alone. In this case, the connections represent signal flow rather than mass flow. Much like in the case of signaling pathways, it is possible to observe a highly complex *crosstalk* between different subpathways. This includes multiple feedback links between upper and lower glycolysis, upper glycolysis and the oxidative part of the pentose-phosphate (PP) pathway, lower glycolysis and the TCA cycle, and a positive feedback link from citrate to upper glycolysis.

Figure 1 shows that most regulatory interactions are inhibitory. It is possible that some of these inhibitory interactions are competitive rather than allosteric (i.e., the binding site of the effector coincides with the catalytic site). Since the binding mechanisms are not generally reported in the databases, and the regulatory effect is similar, this distinction will be disregarded for the purpose of this work.

**Figure 2:**
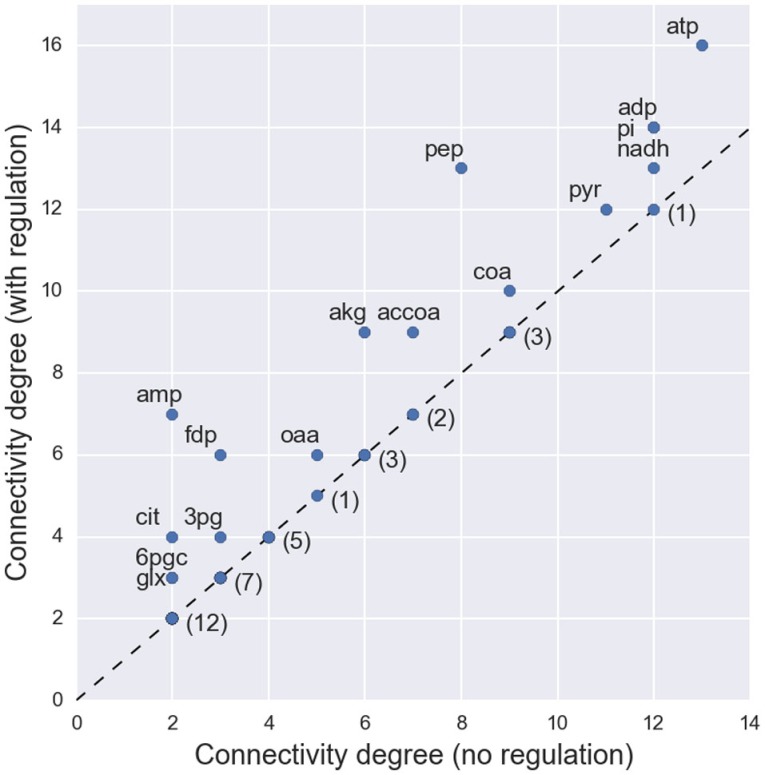
**Changes in the connectivity degree of each metabolite when allosteric regulation is considered in the network topology**. The labeled metabolites represent the cases where the metabolite acts as regulator to a set of enzymes and, consequently, an increase in connectivity is observed. For the nodes with unchanged connectivity only the number of occurrences is presented.

Topological analysis in terms of connectivity degree shows an increased connectivity for several metabolites when allosteric regulation is considered (Figure 2). However, the median value of connectivity remains the same (4 connections per metabolite). Unsurprisingly, there is an increased connectivity for metabolites that were previously known metabolic hubs. For instance, phosphoenolpyruvate (*pep*) is now connected to a total of 13 reactions (previously 8), reinforcing the importance of this glycolytic compound as a metabolic hub (Link et al., 2013; Matsuoka and Shimizu, 2015). However, changes are also observed for lowly connected metabolites. A notable case is *fructose-1,6-bisphosphate* (*fdp*), which can now be considered as a hub metabolite (with a total of 6 connections), although its connectivity is bellow the median if regulation is not considered. This metabolite was recently identified as a key flux-signaling metabolite in the glycolytic flux-sensing mechanism of *E. coli* (Kochanowski et al., 2013b).

### 2.2 Omics data-based analysis of allosteric regulation

In order to understand how the coordination between hierarchical and metabolic regulation drives the metabolic flux, we used the reconstructed model to integrate and analyze a multi-*omics* dataset for *E. coli* (Ishii et al., 2007). This dataset contains transcript, protein, metabolite, and flux data for *E. coli* strains growing aerobically in a chemostat. It comprises several experiments, including variations of dilution rate for the wild-type strain (0.1–0.7 h^−1^) and 24 single knockout mutants growing at the reference dilution rate (0.2 h^−1^). Herein, we will refer to the wild-type strain growing at 0.2 h^−1^ as the reference condition, and the remaining as the perturbed conditions (28 in total).

The data were analyzed using the concept of *regulation analysis* introduced by ter Kuile and Westerhoff (2001) to decompose the contribution of hierarchical (*ρ_h_*) and metabolic (*ρ_m_*) control coefficients during flux change between two experimental conditions (*ρ_h_* + *ρ_m_* = 1). We applied the generalization proposed in Chubukov et al. (2013) to simultaneously compare multiple conditions (see Methods). This generalization assumes that the coefficients are conserved across conditions. The results are presented in Figure S4 in Supplementary Material. It can be observed that in many cases the slopes are close to zero or even negative, indicating poor evidence of transcriptional control. Only three reactions (*PGI*, *CS*, *FUM*) present an estimated hierarchical control coefficient above 0.5. Hence, only these reactions are likely to be predominantly regulated at the transcriptional level.

Given the lack of evident hierarchical control for most enzymes, one can try to analyze the allosteric control exerted by single effectors in a similar fashion (see Methods). The results are presented in Figure S5 in Supplementary Material. In order to observe active flux control, positive slopes would be expected for enzyme activators and negative slopes for enzyme inhibitors. However, this behavior can only be observed in a few cases. The flux of *FBA* positively correlates with its two activators, citrate and *pep*. Some correlation is also observed between ATP levels and two of its inhibition targets, *GND* and *PFK*.

Given the large number of reactions without evident transcriptional or allosteric control, we hypothesize that the assumption of constant control coefficients across all conditions does not hold for the given experimental conditions. It is likely that, during different perturbations, different kinds of control are predominant for each reaction. This has also been observed in previous studies in *S. cerevisiae* (Rossell et al., 2006).

We analyzed the flux change for each reaction at each perturbed condition individually, by comparing the logarithmic change of enzyme, flux, and metabolite levels between all 28 perturbed conditions and the reference condition. Although this would result in a total of 672 potential case studies (24 regulated reactions times 28 perturbations), due to the sparsity of the data (especially the metabolome data), this study was restricted to all reaction-condition pairs with sufficient data to perform a meaningful analysis (see Methods). This reduced the number of case studies to 38 (see Figure S6 in Supplementary Material for details). We then analyzed the evidence of allosteric control for these cases (see Methods) and observed a total of 8 cases where allosteric regulation seems to play a role in controlling the reaction flux for the given perturbation (Figure S6 in Supplementary Material). These 8 cases will be analyzed in detail below.

**Figure 3:**
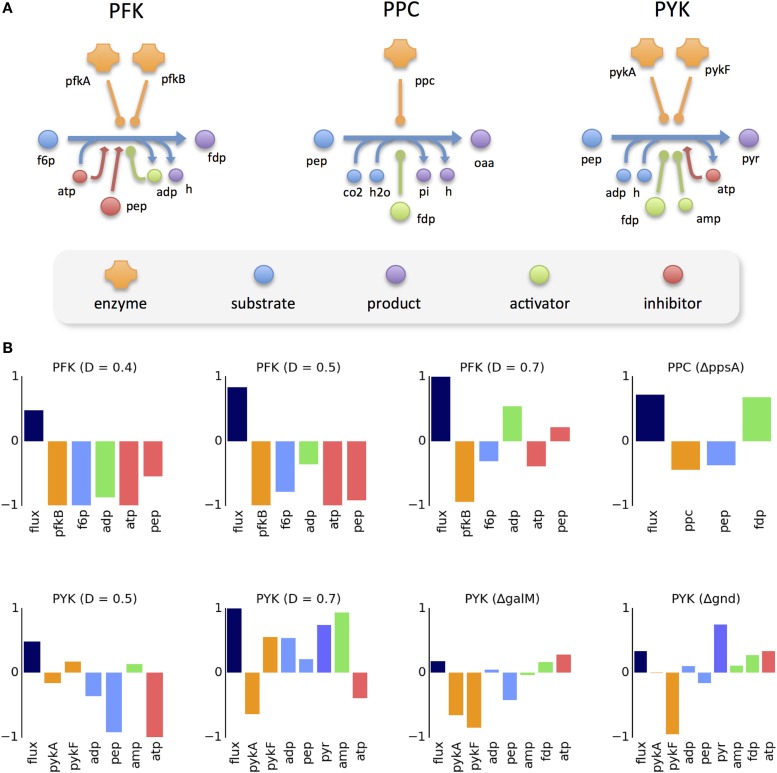
**Data analysis of allosterically regulated reactions**. **(A)** Known regulation mechanism of three reactions analyzed in detail, including all participating molecules. **(B)** Logarithmic change of the metabolic flux and concentrations of the participating molecules between the perturbed and reference condition. Missing proteins and metabolites in the plots correspond to cases where the data were not available.

The regulation mechanisms of the three reactions involved (*PFK*, *PPC*, and *PYK*) are depicted in Figure 3A. The intricate regulation of these enzymes is evident, in particular for *PFK* and *PYK*, which are catalyzed by multiple isozymes and regulated by multiple effectors. The logarithmic change of flux and all measured intervening molecules for the selected reaction-condition pairs is presented (Figure 3B). It can be observed that, in most cases, the change in enzyme concentration is in the opposite direction of the flux change. For *PFK*, only one of the isozymes is measured. In the case of *PYK*, where both isozymes are measured, it can be observed that the level of one isozyme increases while the other decreases. In the few cases where the flux change follows the direction of the enzyme level, the magnitude of enzyme change is still insufficient to explain the flux change (since the reaction rate would be directly proportional to the enzyme concentration). Regarding the change in substrate levels, it can be observed that, in most cases, it is also opposite to the direction of the flux change.

The effect of allosteric control is evident in some scenarios. For instance, in the Δ*ppsA* mutant, the flux of *PPC* largely increases, despite the decrease of its only enzyme (*ppc*) and its main substrate (*pep*). This increase can be explained by the increased concentration of its allosteric activator (*fdp*). There are cases where the different allosteric regulators have a cooperative effect in flux control (e.g., *PYK* at 0.7 h^−1^) and cases where there is a competing effect (e.g., *PFK* at 0.4 h^−1^). One can observe that flux change is not always controlled by the same combination of effectors. For instance, at high dilution rates (0.4–0.5 h^−1^) the flux of PFK increases with the decrease of its inhibitors (ATP and *pep*), despite the decrease of its activator (ADP). However, at an even higher dilution rate (0.7 h^−1^), the flux increase coincides with higher levels of the activator, whereas the two inhibitors change in opposite directions.

The interpretation of the results is hampered by the lack of protein and metabolite measurements for many experimental conditions. One cannot exclude the possibility that some flux changes are also driven by changes in unmeasured isozymes, cofactors, or reaction products.

### 2.3 Model-based prediction of allosteric regulation

Given the scarcity of multi-*omics* datasets with all the data required to perform a quantitative analysis of allosteric regulation, we developed a constraint-based approach for model-based predictions. This method is based on the assumption that, if a reaction is activated (respectively, inhibited) by a compound present in a pathway, then its flux change should be positively (respectively, negatively) correlated with the flux change in that pathway (see Supplementary Material for details). It has been proposed that allosteric intermediates function as flux-signaling metabolites that directly translate flux information to metabolite concentration (Kotte et al., 2010; Matsuoka and Shimizu, 2015). The method, named allosteric regulation FBA (arFBA), is a variation of parsimonious FBA (pFBA) (Lewis et al., 2010) where the objective function is extended as follows:

$$\underset{v}{\min}\;\sum_{i}\;\left|v_{i}\right|+\sum_{R_{ij}>0}\;w_{\mathrm{ij}}\left|\frac{v_{j}}{v_{j}^{0}}-\frac{t_{i}}{t_{i}^{0}}\right|+\sum_{R_{ij}<0}\;w_{\mathrm{ij}}\left|\frac{v_{j}}{v_{j}^{0}}+\frac{t_{i}}{t_{i}^{0}}-2\right|.$$

Here, *v* is the flux distribution to be estimated, *v*^0^ is the flux distribution for a given reference condition, *t_i_* is the turnover rate of metabolite *i*. The allosteric interactions are represented in a new matrix *R*, which has a structure similar to the stoichiometric matrix, with *R_ij_* = 1 (respectively, −1) if metabolite *i* activates (respectively, inhibits) reaction *j*, and 0 otherwise (note that the stoichiometric matrix *S* is not changed). The *w_ij_* parameters are arbitrary weights that represent the strength of the interaction between effector *i* and reaction *j*. If all *w_ij_* are close to zero, then the method defaults to a simple pFBA simulation. The minimization of the extra terms in the objective function affects the respective fluxes when regulation is active. For an activation, the subtraction forces the flux and turnover ratios to be the same. For an inhibition, the term forces that a change in the turnover is compensated by an opposite change in the flux. A detailed justification for these terms is given in the Supplementary Material. The full implementation of the method is slightly more complex due to the presence of reversible reactions and reactions without flux in the reference condition (see Supplementary Material for a complete description).

**Figure 4:**
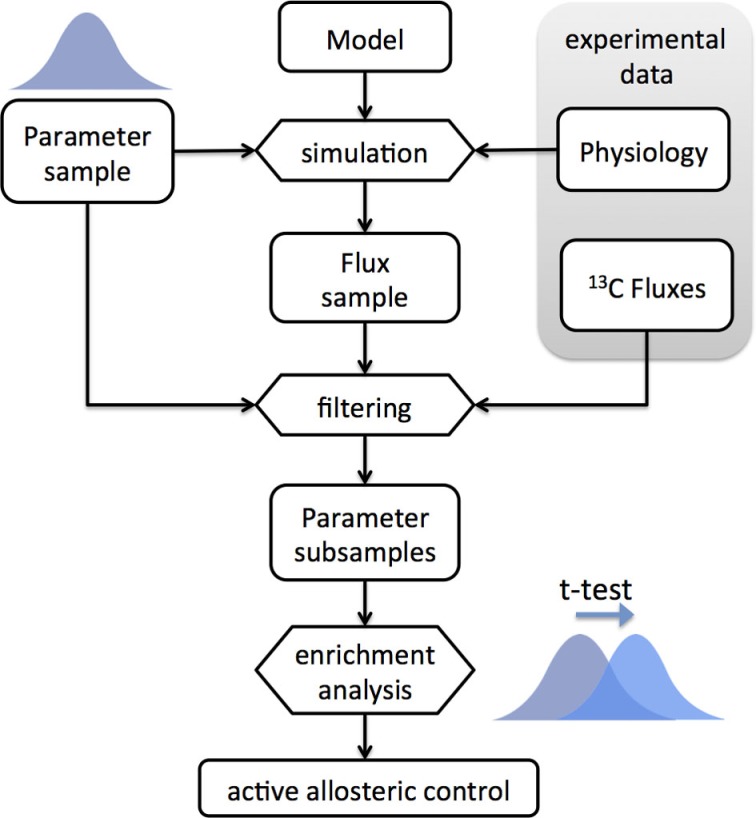
**Workflow diagram of the enrichment analysis based on the ensemble modeling method**. An ensemble of models is built by random sampling of the parameter space (log-normal distribution). Physiological data (growth and uptake rates) are used to constrain the models. The ensemble is used for simulation of flux distributions, which are filtered by comparison with ^13^C-based intracellular flux data. The subset of ensembles with higher predictive ability is compared to those with lower predictive ability and enriched parameters are detected by t-test analysis. The active allosteric control cases are identified by the positively enriched parameters for the respective interactions.

In general, it is not possible to know the strength of the allosteric interactions beforehand. Therefore, we implemented an ensemble modeling approach in order to find the most plausible models (Figure 4). The approach is similar, albeit different, to the ensemble modeling approach used for kinetic modeling (Tran et al., 2008). A model ensemble was built by randomly sampling the *w_ij_* parameters (see Methods). The simulated flux distributions are then compared with the intracellular flux data from Ishii et al. (2007). The accuracy of each model is given by the (*L*_1_-norm) distance between the predicted and measured flux distributions. The original ensemble is split into two groups containing the models with prediction accuracy above and below the median. We then perform enrichment analysis by comparing the distributions of each parameter between the two ensembles. For a particular experimental condition, if a parameter *w_ij_* has systematically higher values in the ensemble with higher predictive accuracy, then the assumption of allosteric control between effector *i* and reaction *j* results in improved flux predictions for that condition.

**Figure 5:**
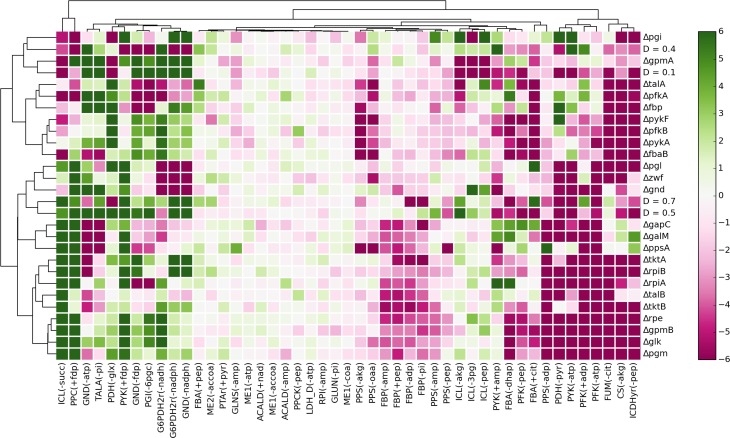
**Enrichment analysis of the parameters associated with each allosteric interaction, represented by the *t*-test value of each parameter subsample for each experimental condition**. The clustering of the heatmap was performed using complete linkage and Manhattan distance.

Figure 5 shows *t*-test values for all parameters across all experimental conditions. Although there are not clearly defined clusters in the clustered heatmap, some general patterns can be observed. About one-quarter of the interactions are positively enriched for most experimental conditions, representing probable cases of active allosteric control for those conditions. On the other hand, almost half of the parameters are negatively enriched for a majority of conditions. These represent allosteric constraints that, in most cases, hamper the predictive ability of the models. Finally, there is a subset of allosteric interactions which are neither positively nor negatively enriched. Accounting for these interactions has very little effect in the prediction of flux distributions for the given experimental conditions.

The most frequent positively enriched interactions include inhibition of the oxidative phase of the pentose-phosphate pathway (PPP) by reducing agents NADH and NADPH; mutual inhibition between PPP and upper glycolysis; feedforward activation of *PPC* and *PYK* by *fdp*; and inhibition of the glyoxylate shunt by multiple effectors. Interestingly, several parameters that are positively enriched for a subset of conditions are also negatively enriched for some of the remaining conditions. Hence, although the respective interactions improve the flux predictions in some conditions, in other conditions they make predictions worse.

In order to test the predictive ability of our *in silico* approach, we analyzed the enrichment results for the potential cases of allosteric control previously detected by data-driven analysis (Figure 3B). Some of the allosteric interactions were significantly enriched, namely the activation of *PFK* by ADP at the highest dilution rate (*t* = 4.28, *p* = 1.88e-5), activation of *PPC* by *fdp* in the Δ*ppsA* mutant (t = 19.0, *p* = 8.17e-79), and activation of *PYK* by *fdp* in the Δ*gnd* mutant (*t* = 4.70, *p* = 2.63e-6) and the Δ*galM* mutant (*t* = 7.09, *p* = 1.44e-12).

**Figure 6:**
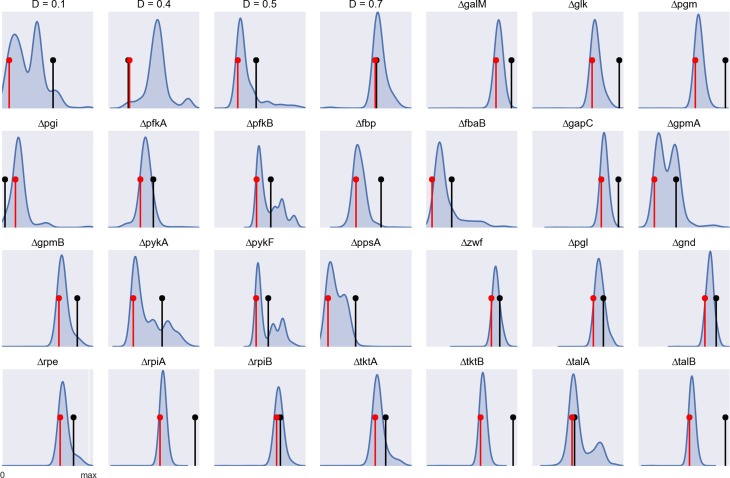
**Simulation accuracy determined by the (*L*_1_-norm) distance between experimental and simulated flux distributions for each experimental condition (data normalized by the maximum distance value)**. The blue curve shows the frequency distribution of the distances obtained by random sampling of the weighting factors in arFBA. The black pin marks the distance obtained with an FBA simulation. The red pin marks the distance obtained with arFBA after calibration of the weighting factors.

It should be noted that we are using our simulation method (arFBA) in the reverse direction, i.e., a model ensemble is compared with experimental data to find which parameters (weighting factors) result in improved predictions. Although, in theory, one could use the method in the forward direction, i.e., to perform simulations with improved flux predictions, this would require finding a “universal” parameter configuration that fits all conditions. The previous results show that such universal configuration cannot be found due to the condition-specific nature of allosteric regulation. Nonetheless, we tested the accuracy of arFBA by measuring the distance between simulated and experimental flux distributions. Figure 6 shows the frequency distribution of the distances obtained by random sampling of the weighting factors for each experimental condition. The distance obtained with FBA is shown for comparison. It can be observed that, for most experimental conditions, the average distance obtained with arFBA is lower than that obtained with FBA, indicating a higher accuracy of the former. Finally, we tested the accuracy of arFBA with *a posteriori* calibration of the weighting factors (see methods). It can be observed that, after calibration, the accuracy of arFBA is higher than FBA for 26 of the 28 conditions.

## 3 Discussion

In this work, we analyzed the role of allosteric regulation for flux control in the central carbon metabolism of *E. coli*. For this, we extended a constraint-based metabolic model of *E. coli* with allosteric regulation. The application of such a model is twofold. First, it can be used as an integrative scaffold for multi-*omics* dataset analysis, revealing the coordination between enzyme, metabolite, and flux levels. Second, it can be used for *in silico*-based predictions that account for allosteric regulation in the simulation of the metabolic phenotype. For that purpose, we implemented an FBA variant, named arFBA, that accounts for allosteric interactions in the determination of the flux distribution.

Using the expanded model and a multi-*omics* dataset for *E. coli* (Ishii et al., 2007), we analyzed the impact of allosteric regulation in controlling the metabolic flux under multiple environmental and genetic perturbations. We implemented a generalized form of regulation analysis (ter Kuile and Westerhoff, 2001) in order to find which reactions are predominantly under transcriptional or allosteric control. The results reveal that most reactions are generally not controlled by the same mechanism across all conditions. This led us to analyze the effects of perturbations in single reactions for each experimental condition. This analysis is hampered by missing protein and metabolite measurements, which does not allow accounting for all participating compounds in the reactions analyzed. Although we neglected the effect of missing isozyme and cofactor measurements, as well as product concentrations for irreversible reactions, only 38 out of 672 possible case studies (24 reactions × 28 perturbations) could be analyzed in a meaningful way (Figure S6 in Supplementary Material). Nonetheless, it was possible to identify 8 (out of 38) cases where the reaction flux is predominantly controlled by allosteric mechanisms.

Considering that the dataset published by Ishii et al. (2007) is one of the most comprehensive multi-*omics* dataset for a model organism published so far, we can conclude that purely data-driven analysis is very limited for studying metabolic regulation. Therefore, we applied our simulation method using an ensemble modeling approach to identify which allosteric interactions result in improved flux predictions. Enrichment analysis of the weighting factors in our model revealed that several allosteric interactions were significantly enriched when the models were filtered by their agreement with experimental flux data. A comparison between the *in silico* results and the data-driven analysis showed that 4 of the 8 cases of allosteric control previously identified were also detected by the computational approach.

Given the very limited scope of the cases analyzed in detail, the cross-comparison between the data driven and *in silico* results can hardly be considered a validation of the latter. In order to determine the accuracy of the simulation method it would be necessary to estimate the number of false positive and false negative results for the whole dataset. Instead, the two approaches should be seen as complementary methods to guide the analysis of allosteric regulation. Furthermore, the data analysis revealed that the predominant mode of regulation for each reaction is condition dependent. This was also observed in the *in silico* analysis, hampering the determination of a universal set of weighting factors for arFBA. Given the interplay between different regulation mechanisms, the approach developed herein could be suitable for integration with other methods for identification of regulation mechanisms (Bordel et al., 2010).

An ensemble modeling approach was also employed by Link et al. (2013) for systematic identification of allosteric interactions in *E. coli*. The authors measured metabolite concentrations using rapid sampling and ^13^*C*-labeled substrates (glucose and fructose) to determine the transient profile of glycolytic intermediates in dynamic cultures switching between glycolysis and gluconeogenesis. A kinetic ensemble model for glycolysis was used to test 126 putative interactions. The results not only confirmed previously known interactions but also predicted new interactions that had not been previously reported. Although the model used in this study differs from ours, the results regarding interactions common to both models are consistent. In particular, both studies revealed the importance of *PFK* as an active regulation target for controlling the glycolytic flux, and the role of *fdp* as key regulator of *PPC* and *PYK* to control *pep* consumption.

At the end of our data-driven analysis, some flux changes remain unexplained by hierarchical or metabolic control. One main reason for this is the lack of coverage of the metabolomics data, which only accounts for approximately half of the metabolites in the model. Another possibility is that the regulatory mechanisms for the respective enzymes are not fully known or the relevant allosteric interactions were not included in the model. It is also possible that the enzyme concentrations do not correlate with the respective enzymatic activity due to post-translational modifications (PTMs). It has been shown that PTMs, such as acetylation, have important regulatory functions in *E. coli* (Castaño-Cerezo et al., 2014).

The generation of high-quality multi-*omics* datasets will be necessary for a deeper understanding of metabolic regulation. Herein, we used a previously published dataset for chemostat cultures. However, steady-state data may be insufficient to analyze regulatory responses. It has been observed that fast metabolic responses precede the slower transcriptional response during metabolic adaptation (Ralser et al., 2009). Since allosteric regulation operates on a faster time-scale compared to transcriptional regulation, transient profiles on short time scales should be particularly informative (Link et al., 2013).

## 4 Conclusion

In this work, we focused on the role of allosteric regulation in central carbon metabolism. The reconstruction of an allosteric model revealed that allosteric information is inconsistent among different data sources even for these highly studied pathways. The allosteric interactions added a new layer to the network topology, changing the overall network connectivity and revealing metabolic hubs that would otherwise be ignored (e.g., *fdp*). Hierarchical and allosteric regulation analysis using a multi-*omics* dataset revealed that there is no predominant mechanism of regulation across all experimental conditions. Nonetheless, situations of predominant allosteric control could be identified for some reactions at particular conditions. Our new method for model-based prediction of allosteric control was able to capture at least a few of these situations. However, the assessment of the predictive ability of this method is hampered by the lack of more comprehensive data.

For central carbon metabolism, it would have been feasible to perform this analysis using a kinetic modeling approach [similarly to Link et al. (2013)]. However, as we move toward regulatory analysis at the genome-scale, the constraint-based approach should become especially useful. Building a genome-scale model of allosteric regulation is a daunting task that will require literature mining, extensive manual curation, and prediction of putative interactions. Our knowledge of the *allosterome* is currently limited by the lack of high-throughput screening methods for detecting metabolite–enzyme interactions. It is likely that the vast majority of allosteric interactions are yet to be discovered (Lindsley and Rutter, 2006). Recent experimental methods have been developed toward systematic identification of metabolite-protein interactions (Gallego et al., 2010; Li et al., 2010; Orsak et al., 2011; Feng et al., 2014). However, we are still far from a genome-scale screening of the hundreds of thousands of potential interactions between all metabolites and enzymes in an organism.

Notebaart et al. (2014) have recently unraveled the *underground* metabolism of *E. coli* by expanding a genome-scale metabolic model with reactions resulting from promiscuous enzyme activity. With the *allosterome*, we can unravel yet another hidden layer in the network topology of cellular metabolism. New expanded models of metabolism will be certainly useful for applications, such as drug discovery and rational strain design, as we slowly move toward what has been called the “second secret of life” (Fenton, 2008).

A python implementation of arFBA as well as the allosteric model in SBML format are available on GitHub: https://github.com/cdanielmachado/arfba.

## 5 Materials and Methods

### 5.1 Model reconstruction

The original model of the core metabolism of *E. coli* (Orth et al., 2009) was extended with allosteric interactions obtained from BRENDA (Schomburg et al., 2002), EcoCyc (Keseler et al., 2011), and two previously published kinetic models (Chassagnole et al., 2002; Kotte et al., 2010). We searched for evidence of regulatory interactions for each possible combination of enzymes and metabolites in the model. A total of 148 regulatory interactions were found (Figure S3 in Supplementary Material). Since the majority of these interactions can only be found in one data source, for the sake of curation we only included in the model the interactions that are reported in at least two different sources. In a few cases the same metabolite is reported as activator and inhibitor of an enzyme (e.g., *phosphoenolpyruvate* binding to *fructose-bisphosphatase*). In these cases, we used the most frequently reported effect.

### 5.2 Regulation analysis

#### 5.2.1 Cross-Condition Analysis

The metabolic flux of a reaction (*J_i_*) can be generically described in terms of the concentrations of the respective enzyme(s) (*E_i_*) and all the intervening metabolites (substrates, products, effectors):

$$J_{i}=k_{\text{cat}}E_{i}f\left(M\right)$$

where *k*_cat_ is the turnover rate of the enzyme, and *f* (*M*) represents a non-linear function of the metabolite concentrations. *Regulation analysis* introduced by ter Kuile and Westerhoff (2001) decomposes the contribution from hierarchical and metabolic control by considering the logarithmic change between two experimental conditions:

$$\Delta \log\left(J_{i}\right)=\Delta \log\left(E_{i}\right)+\Delta \log\left(f\left(M\right)\right)$$

and estimating the respective contribution coefficients:

$$1=\frac{\Delta \log\left(E_{i}\right)}{\Delta \log\left(J_{i}\right)}+\frac{\Delta \log\left(f\left(M\right)\right)}{\Delta \log\left(J_{i}\right)}=\rho_{h}+\rho_{m}.$$

Since *f* (*M*) is generally unknown, one can estimate *ρ_h_* (and consequently *ρ_m_*) by measuring the enzyme and flux levels across different conditions. Chubukov et al. (2013) generalized this comparison from two to multiple conditions in order to decrease the effects of experimental error. The estimation is performed by linear regression between log(*E_i_*) and log(*J_i_*) across all experimental conditions using a robust linear regression method (Theil–Sen estimator).

We further generalized this concept to the study of allosteric regulation, by decoupling the effect of allosteric regulators in the reaction flux from the non-linear *f* (*M*) component, using a power-law approximation:

$$f\left(M\right)\approx g\left(S,P\right)\prod_{j}\;A_{j}^{\gamma_{\mathrm{ij}}}\prod_{j}\;I_{j}^{-\gamma_{\mathrm{ij}}}$$

where *S*, *P*, *A*, *I* represent, respectively, the set of substrates, products, activators and inhibitors of reaction *i*, and *γ_ij_* is the apparent kinetic order of effector *j* in reaction *i*, as defined in Biochemical-Systems Theory (Voit, 2013). This allows us to estimate individual allosteric regulation coefficients (*ρ_a_*) for each effector as:

$$\rho_{\text{a}\left(j\right)}=\left\{\begin{array}{cc} \gamma_{\text{ij}}\frac{\Delta \log\left(A_{j}\right)}{\Delta \log\left(J_{i}\right)}\; & \text{if}\;j\;\text{is}\;\text{an}\;\text{activator}\;\text{of}\;i\\ -\gamma_{\text{ij}}\frac{\Delta \log\left(I_{j}\right)}{\Delta \log\left(J_{i}\right)}\; & \text{if}\;j\;\text{is}\;\text{an}\;\text{inhibitor}\;\text{of}\;i \end{array}\right.$$

With the exception of effectors exhibiting cooperative binding, we can assume that the kinetic orders are close to or below unity (*γ_ij_* ≤ 1). Hence, the allosteric control coefficient is bound by the slope of the linear regression.

Regulation analysis was performed for all allosterically regulated reactions with available fluxomics and proteomics data. A total of 18 (out of 24) regulated reactions were experimentally measured. Due to gaps in the proteomics dataset, we restricted the analysis to enzymes with available data for at least 10 (out of 29) experimental conditions.

#### 5.2.2 Single-Condition Analysis

Allosteric effects were analyzed for each perturbation individually by comparing the logarithmic change of enzyme, flux, and metabolite levels between all 28 perturbed conditions and the reference condition. Due to the sparsity of the data (especially the metabolome data), this analysis was restricted to all reaction-condition combinations where the following criteria were satisfied: (1) at least one associated enzyme was measured; (2) all main substrates (excluding cofactors) were measured; (3) at least one effector was measured. Furthermore, we excluded flux changes that were not significant (i.e., the perturbed flux falls within a 95% confidence interval of the reference flux).

Evidence of allosteric control was detected by selecting conditions where the flux change is not fully explained by changes in enzyme concentration (Δlog(*E*)/Δlog(*J*) < 0.5) or substrate abundance (Δlog(*S*)/Δlog(*J*) < 0.5), and is at least partly related with changes in one allosteric activator (Δlog(*A*)/Δlog(*J*) > 0.25) or inhibitor (−Δlog(*I*)/Δlog(*J*) > 0.25). For reversible reactions, the effect of flux changes arising from changes in the thermodynamic driving force cannot be excluded. Therefore, for these reactions we only considered reactions where the products were experimentally measured (excluding cofactors) and the flux change cannot be fully explained by the change in product abundance (−Δlog(*P*)/Δlog(*J*) < 0.5).

#### 5.2.3 Ensemble Modeling with arFBA

For each experimental condition, an ensemble of 10^4^ models was built by sampling the weighting factors (*w_ij_* parameters) from a log-normal distribution. Each model is constrained with the experimentally measured glucose and oxygen uptake rates, and the growth rate, which is given by the dilution rate.

#### 5.2.4 Calibration of Weighting Factors in arFBA

Condition-specific weighting factors were calibrated for each experimental condition as follows: an ensemble of 10^4^ arFBA models was built as described above; the accuracy of each model was determined by the *L*_1_-norm distance between the experimental and simulated flux distributions; the calibrated weighting factors were calculated as the average of the 10% most accurate models.

## Conflict of Interest Statement

The authors declare that the research was conducted in the absence of any commercial or financial relationships that could be construed as a potential conflict of interest.

## Supplementary Material

The Supplementary Material for this article can be found online at http://journal.frontiersin.org/article/10.3389/fbioe.2015.00154
