# Supplementary material for: Modeling the Contribution of Allosteric Regulation for Flux Control in the Central Carbon Metabolism of E. coli
Source: Front Bioeng Biotechnol. 2015 Oct 8;3:154. doi: 10.3389/fbioe.2015.00154 (PMC4597111; doi:10.3389/fbioe.2015.00154)
Supplement: Supplementary file 1 [file DataSheet_1.PDF]

Supplementary Material:
Modeling the contribution of allosteric regulation for flux control in the central carbon metabolism of *E. coli*

Daniel Machado*, Markus J. Herrgård and Isabel Rocha
*Correspondence:
Daniel Machado
dmachado@deb.uminho.pt

1 SUPPLEMENTARY TEXT

1.1 Effect of turnover rates

Since constraint-based models do not account for metabolite concentrations, we used the metabolite turnover rate as a proxy for its concentration. Although the concentration of a metabolite at steady-state is not directly proportional to its turnover rate, we can try to estimate how the flux of a regulated reaction is affected by the turnover rate of the respective effector. For this purpose, we built two kinetic toy models of metabolic systems exhibiting activation and inhibition (Fig. S1 and Table S1).

The external concentration of effector X was varied by several orders of magnitude and the steady-state flux distribution was calculated for each value. We analyzed how the flux of the activated and inhibited reactions varied with respect to the change in the turnover rate of the effector (Fig. S2). We also varied the value of the activation and inhibition constants in order to observe their effect in the sensitivity of the reaction rate to the turnover rate. We can observe that for intermediate values of these constants, the response is close to linear.

1.2 Constraint-based modeling of allosteric effects

Following the previous results, we will assume that the effect of the turnover rate of an effector (i) in the flux of an activated reaction (j) is approximated by $v_j/v_j^0 = t_j/t_j^0$, and the effect on the flux of an inhibited

Supplementary Table 1. Kinetic rate laws and kinetic parameters used in the toy models.

	Kinetic rate law	Kinetic parameters
R_1	$V_{\max}\frac{S}{K_M+S}\frac{X}{K_A+X}$	$V_{\max}=1,\ K_M=1,\ K_A=1$
R_2	$V_{\max}\frac{S}{K_M+S}\frac{1}{1+X/K_I}$	$V_{\max}=1,\ K_M=1,\ K_I=1$
In_S	$k_t(S_{\text{ext}}-S)$	$k_t=1,\ S_{\text{ext}}=10$
In_X	$k_t(X_{\text{ext}}-X)$	$k_t=1,\ X_{\text{ext}}=1.5$
Out_P	$V_{\max}\frac{P}{K_M+P}$	$V_{\max}=1,\ K_M=1$
Out_X	$V_{\max}\frac{X}{K_M+X}$	$V_{\max}=1,\ K_M=1$

reaction (j) is approximated by $v_j/v_j^0 = 2 - t_j/t_j^0$. This approximation is used to implement the objective function of our method, allosteric regulation FBA (arFBA), which is implemented as follows:

$$
\begin{aligned}
\min_{v} \; & \sum_{j \in Irrev} v_j + \sum_{j \in Rev} \left(v_j^f + v_j^r \right) \\
& + \sum_{R_{ij}>0, v_j^0 \neq 0, t_i^0 \neq 0, j \in Irrev} w_{ij} \left| \frac{v_j}{v_j^0} - \frac{t_i}{t_i^0} \right| \\
& + \sum_{R_{ij}>0, v_j^0 \neq 0, t_i^0 \neq 0, j \in Rev} w_{ij} \left| \frac{v_j^f + v_j^r}{v_j^{f^0} + v_j^{r^0}} - \frac{t_i}{t_i^0} \right| \\
& + \sum_{R_{ij}<0, v_j^0 \neq 0, t_i^0 \neq 0, j \in Irrev} w_{ij} \left| \frac{v_j}{v_j^0} + \frac{t_i}{t_i^0} - 2 \right| \\
& + \sum_{R_{ij}<0, v_j^0 \neq 0, t_i^0 \neq 0, j \in Rev} w_{ij} \left| \frac{v_j^f + v_j^r}{v_j^{f^0} + v_j^{r^0}} + \frac{t_i}{t_i^0} - 2 \right| \\
\text{s.t} \; & \\
& S \cdot v = 0 \\
& 0 \leq v \leq ub \\
& v_{\text{growth}} = v_{\text{growth}}^* \\
& t_i = \sum_{S_{ij}>0} S_{ij} v_j \\
& v_j^f \leq y_j^f \cdot M \quad \forall j \in Rev \\
& v_j^r \leq y_j^r \cdot M \quad \forall j \in Rev \\
& y_j^f, y_j^r \in \{0, 1\} \quad \forall j \in Rev \\
& y_j^f + y_j^r \leq 1 \quad \forall j \in Rev
\end{aligned}
$$

where v is the flux distribution for the perturbed condition to be estimated, v^0 is the flux distribution for the reference condition, v_j^f and v_j^r are decomposed reversible reactions, t_i is the turnover rate of metabolite i, R_{ij} is the regulatory matrix, w_{ij} are weighting factors, and v_{growth}^* is the measured growth rate for the perturbed condition.

2 SUPPLEMENTARY FIGURES

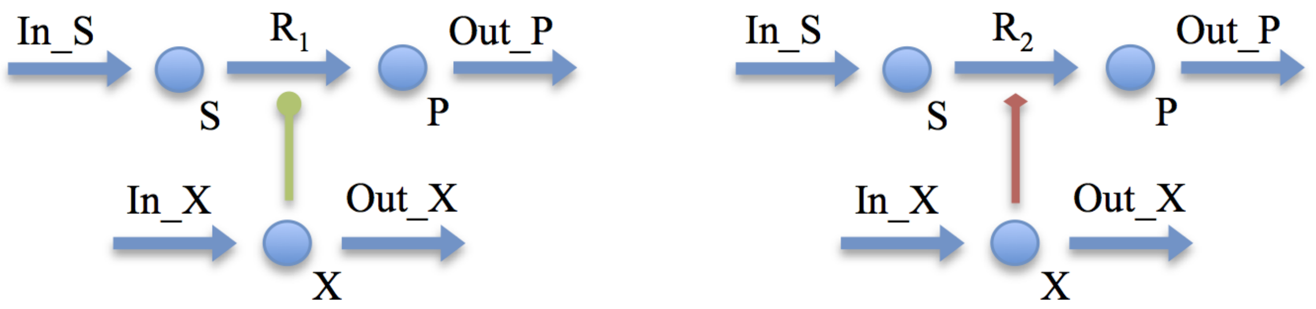

Supplementary Figure 1. Simple toy models of metabolic reaction networks exhibiting activation and inhibition, respectively.

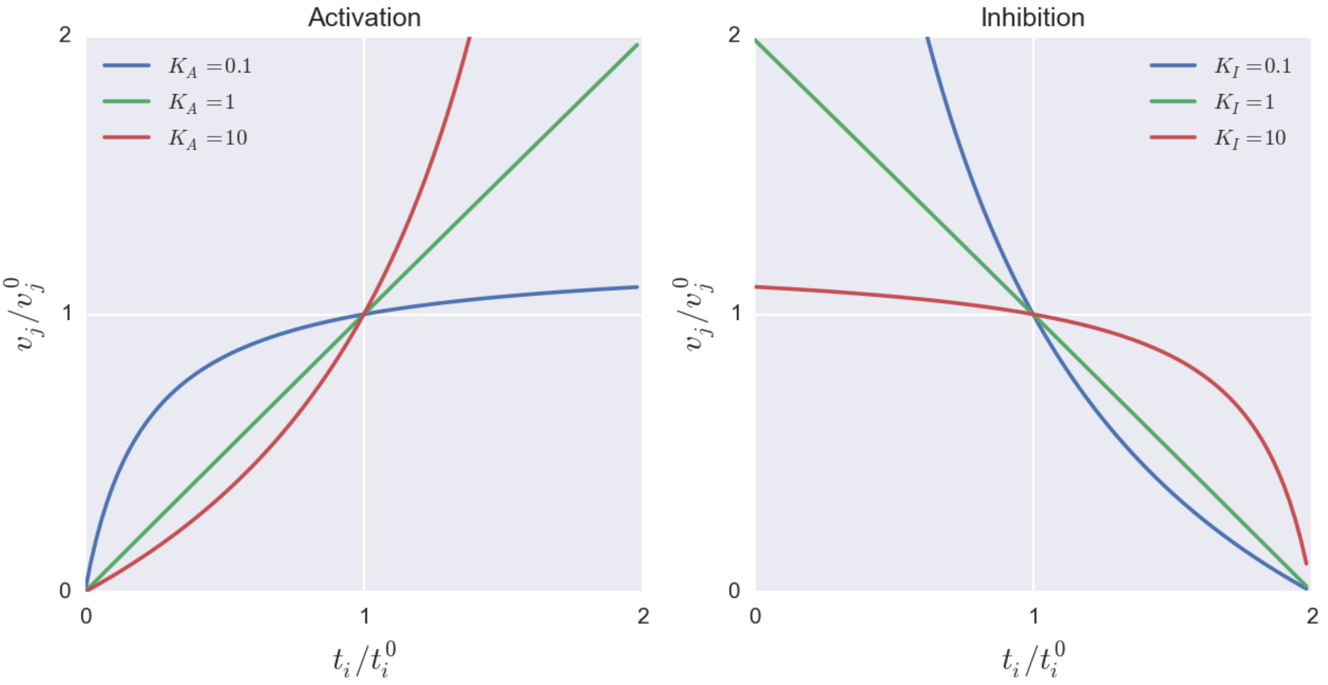

Supplementary Figure 2. Variation of the reaction flux (v_j) of the activated (R_1) and inhibited (R_2) reactions at steady-state as a function of the variation of the turnover rate (t_i) of effector X.

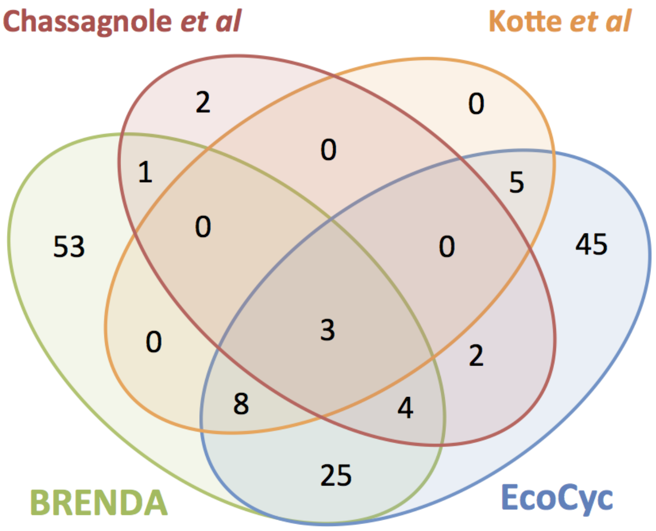

Supplementary Figure 3. Comparison of the number of allosteric interactions retrieved from the different data sources, including the two databases, BRENDA and EcoCyc, and the kinetic model reconstructions from Chassagnole *et al*, 2002 and Kotte *et al*, 2010.

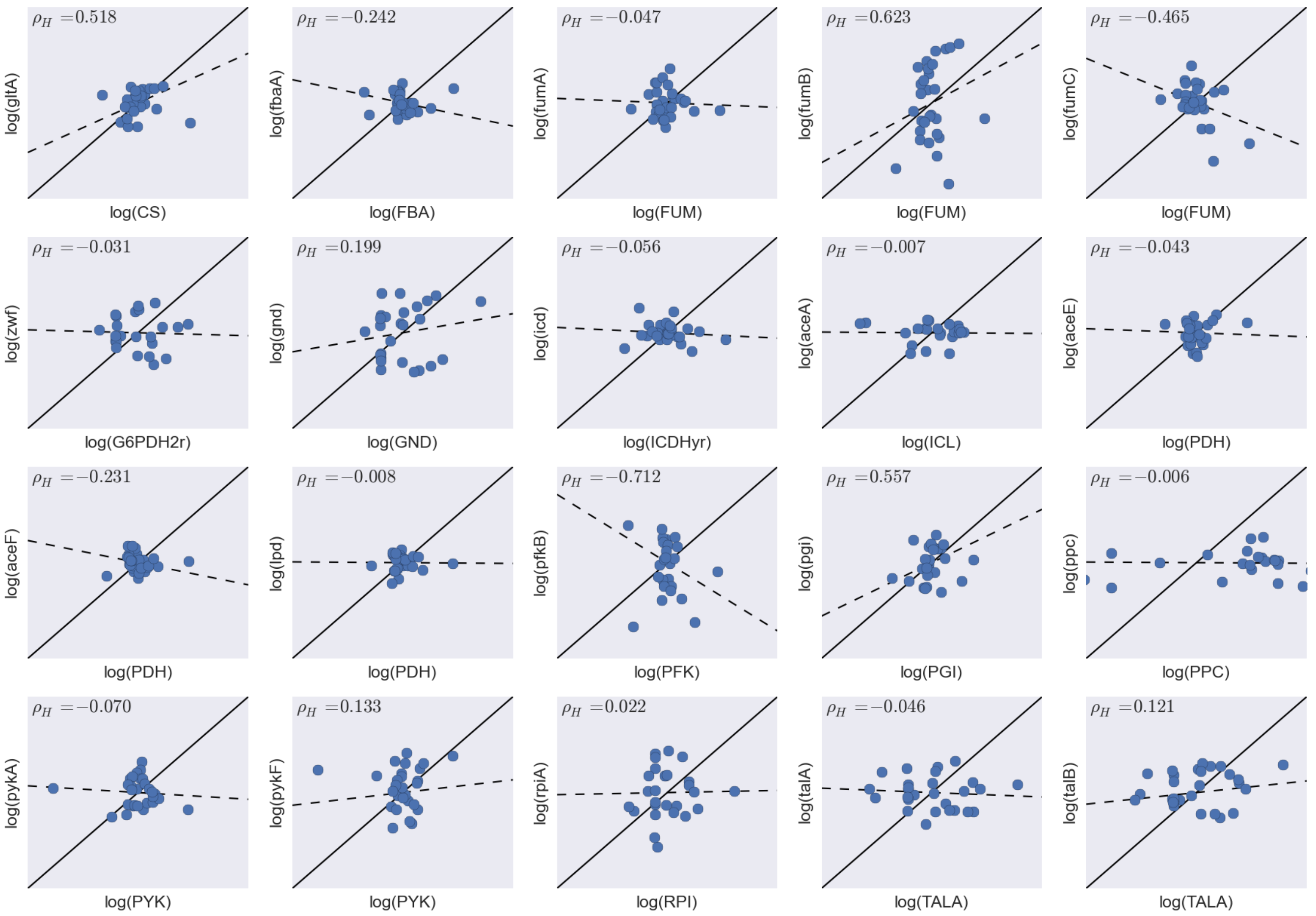

Supplementary Figure 4. Estimation of the hierarchical control coefficients (ρ_h) for all the selected enzyme-reaction pairs using the Theil-Sen slope estimation method. The enzyme concentrations (nmol/gDW) and reaction fluxes (mmol/gDW/h) are log-scaled and normalized by subtracting the respective means. All plot ranges are [-3, 3] in both axis.

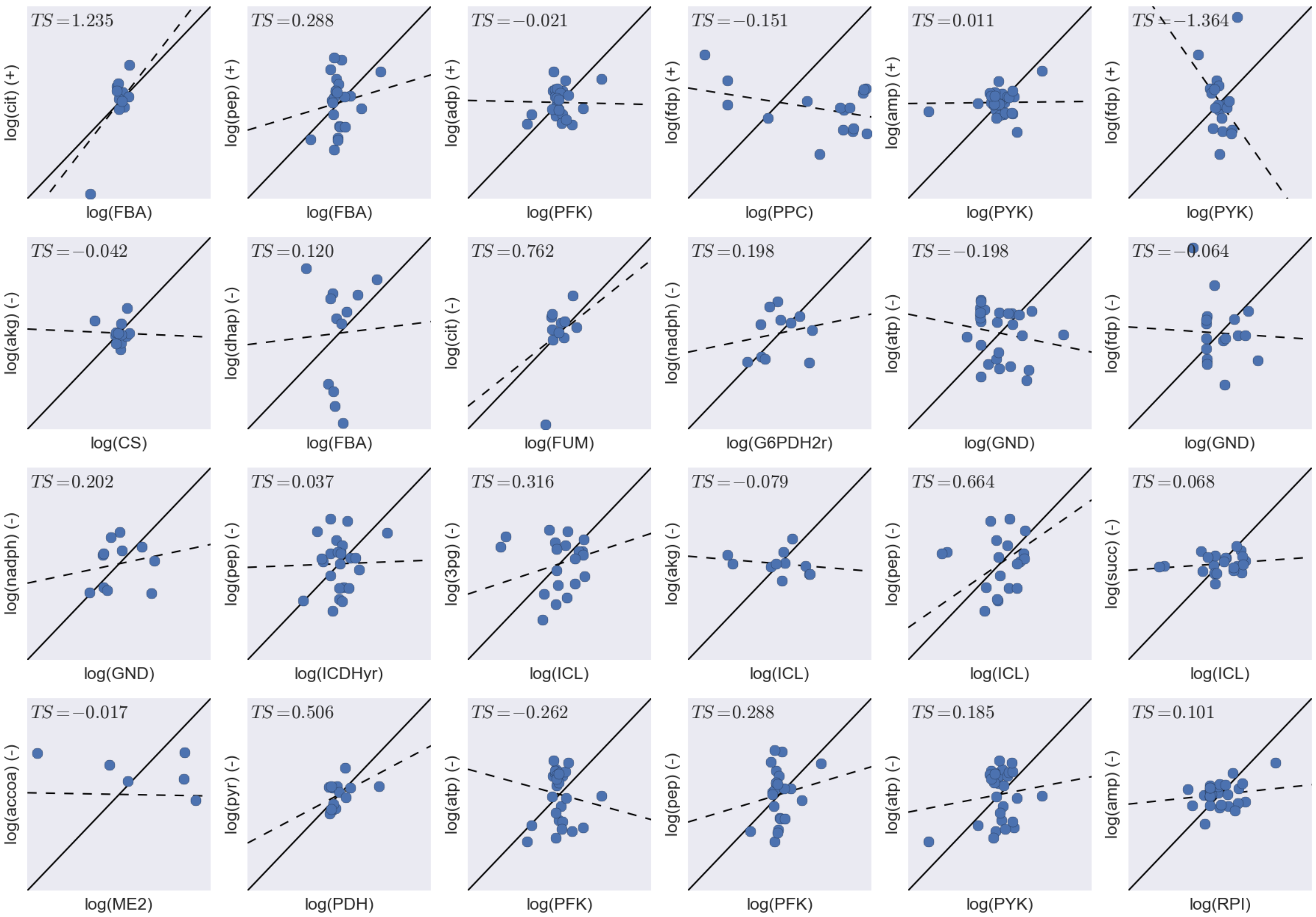

Supplementary Figure 5. Estimation of the Theil-Sen slopes for multiple effector-reaction pairs. The metabolite concentrations (mM) and reaction fluxes (mmol/gDW/h) are log-scaled and normalized by subtracting the respective means. All plot ranges are [-3, 3] in both axis. For the sake of readability, a few outliers are not visible. Nevertheless, they have little influence in the results due to the robustness of the Theil-Sen estimator.

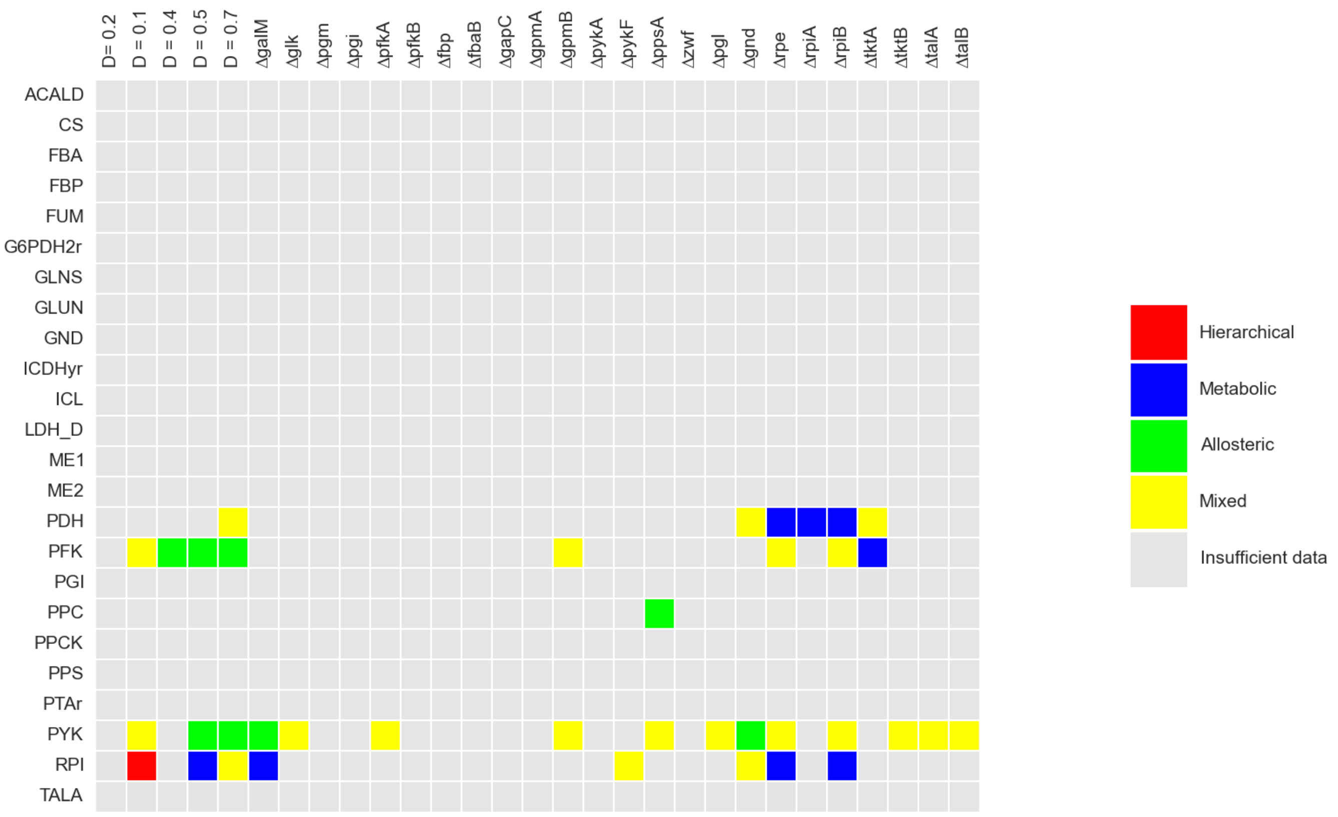

Supplementary Figure 6. Overview of the potential case studies for analyzing the flux control of all allosterically regulated reactions across all the perturbed conditions, including changes in dilution rates (D) and single gene mutants. For the vast majority of case studies (672 in total) there is insufficient data (at flux, protein or metabolite levels) to estimate the contribution of each kind of control. For the 38 remaining cases the main type of control is identified (hierarchical, metabolic, allosteric or mixed control).
